# Post-acute sequelae of COVID-19: A metabolic perspective

**DOI:** 10.7554/eLife.78200

**Published:** 2022-03-23

**Authors:** Philipp E Scherer, John P Kirwan, Clifford J Rosen

**Affiliations:** 1 Touchstone Diabetes Center University of Texas Southwestern Dallas United States; 2 Pennington Biomedical Research Center Baton Rouge United States; 3 Center for Clinical and Translational Research, Maine Medical Center Research Institute Scarborough United States; Icahn School of Medicine at Mount Sinai United States; Icahn School of Medicine at Mount Sinai United States

**Keywords:** COVID-19, PASC, metabolic dysfunction, obesity

## Abstract

The SARS-CoV-2 pandemic continues to rage around the world. At the same time, despite strong public health measures and high vaccination rates in some countries, a post-COVID-19 syndrome has emerged which lacks a clear definition, prevalence, or etiology. However, fatigue, dyspnea, brain fog, and lack of smell and/or taste are often characteristic of patients with this syndrome. These are evident more than a month after infection, and are labeled as *Post-Acute Sequelae of CoV-2* (PASC) or commonly referred to as long-COVID. Metabolic dysfunction (i.e., obesity, insulin resistance, and diabetes mellitus) is a predisposing risk factor for severe acute COVID-19, and there is emerging evidence that this factor plus a chronic inflammatory state may predispose to PASC. In this article, we explore the potential pathogenic metabolic mechanisms that could underly both severe acute COVID-19 and PASC, and then consider how these might be targeted for future therapeutic approaches.

## Introduction

Since January 2020 nearly 600 million people have been infected worldwide with the SARS-CoV-2 virus, the vast majority of whom have developed COVID-19, the disease (A Sehgal et al., 2021). COVID-19 has also resulted in nearly 6 million deaths, primarily as a result of pulmonary injury and its subsequent comorbidities. Despite the availability of several vaccines and boosters, the pandemic has continued, and the cost to society continues to mount (Ahmad et al., 2020b). The most recent viral variant in early 2022, omicron, rapidly spread across virtually every country, with significant morbidity and mortality, coupled with a greater degree of infectivity. Although the majority of individuals infected with SARS-CoV-2 recover, there is a significant subpopulation of patients with persistent symptoms 4 weeks after infection, even among those who suffered from mild-to-moderate COVID-19. Some of that symptomatology include brain fog, exertional fatigue, dyspnea, chest tightness, gastrointestinal disturbances, and vertigo (Alkodaymi et al., 2022). Those symptoms and their resultant clinical signs (e.g., glucose intolerance, sleep disorders, postural hypotension, and cardiac arrythmias) may also represent prodromes to future health threats (e.g., development of autoimmune diseases). The demographics of individuals who suffer from these complaints are insufficient to draw conclusions about gender, age, and social determinants of health as contributing factors (Bechmann et al., 2022). The term *Post-Acute Sequelae of CoV-2* or PASC is a label for those who suffer from symptoms after COVID-19, although the lay terminology ‘long-COVID’ is often used interchangeably. However, because the clinical spectrum of COVID-19 continues to evolve due to differences in substrains, vaccines, and susceptibility by ethnicity and race, it is extremely challenging to define post-COVID-19 syndromes. However, there are clues that metabolic dysfunction may promote or enhance these syndromes. Nevertheless, only by collecting the best evidence about pathogenesis can we hope to understand the temporal aspects of this postviral syndrome, and design the most appropriate treatment approaches. In this review, we will first examine the role of hyperglycemia and obesity in the underlying pathophysiology of severe COVID-19 and then set the stage for characterizing the relationship between post-COVID-19 symptoms and underlying metabolic dysfunction.

## Definitions

The prevalence of PASC is unknown, and the estimates differ by geographic location, timing of illness, duration, ethnicity, racial composition, sex, viral variant, and vaccination status. Current estimates vary widely; somewhere between 12% and 50% of individuals who are infected with SARS-CoV-2 will likely develop post-COVID symptoms more than a month after an initial infection (Bechmann et al., 2022). Some, but not all investigators believe that PASC is a clear-cut postviral syndrome associated with SARS-CoV-2 similar in onset and duration to other postinfective conditions. For this report, we define an acute SARS-CoV-2 infection as COVID 19, confirmed by a positive nasal or throat PCR test or positive nucleocapsid antibody test. In respect to PASC, we limit the term to symptomatology that persists more than 1 month after an acute infection that is confirmed by a positive PCR nasal or throat swab for SARS-CoV-2. It should be noted there are many asymptomatic infections often not included in current epidemiologic estimates, and this is particularly true for omicron infections. These missing cases are relevant when considering the overall incidence of PASC, as well as the ensuing clinical course. Unfortunately, without widespread use of nucleocapsid antibodies, the magnitude of that component of the pandemic will likely remain unknown.

Abbreviations: SARS-CoV-2: the virus responsible for the illness COVID-19; PASC: Post-Acute Sequelae of CoV-2; T2D: type 2 diabetes mellitus; T1D: type 1 diabetes mellitus; BMI: body mass index; GLP-1: glucagon like peptide-1; DPP-4: dipeptidyl peptidase-4; TMPSSR2: transmembrane protease serine-2 precursor; PPARγ: peroxisome proliferator activated receptor gamma.

## Metabolic dysfunction and acute SARS-CoV-2

### Epidemiology of glucose intolerance and acute infection

PASC may be distinct in its pathogenesis from acute SARS-CoV-2 infection, but it is relevant to consider the underlying metabolic factors that contribute to the severity of the initial infection, particularly if viral persistence and chronic inflammation in adipose depots or elsewhere is an underlying risk for prolonged symptomatology. There is still debate as to whether obesity with or without insulin resistance and type 2 diabetes (T2D), is associated with a greater risk of being infected with the SARS-CoV-2 virus. But it is clear these conditions are established risk factors for the development of severe COVID-19 suggesting a predisposition for enhanced viral entry into cells of the respiratory tract (Aminian et al., 2021; Yang et al., 2021; Drucker, 2021). Mendelian randomization studies have analyzed potential causal associations linking 17 cardiometabolic risk factors, including body mass index (BMI), with susceptibility to severe SARS-CoV-2 infection although confounding from T2D cannot be excluded (Leong et al., 2020). Nevertheless, hyperglycemia, with or without a history of diabetes, is a strong predictor of in-hospital adverse outcomes, portending a sevenfold higher mortality compared to patients with well-controlled blood glucose levels (Zhu et al., 2020). Analysis of COVID-19 mortality in participants from the United Kingdom (UK) BioBank showed that individuals with higher BMI, had an increased risk of COVID-19-related mortality (Peters et al., 2020). This is especially pertinent for patients in the ICU infected with SARS-CoV-2, in whom higher BMI is strongly associated with greater risk of mortality (Cummings et al., 2020).

The underlying pathophysiology behind these metabolic risks are less clear although glucose intolerance from the stress of infection, coexistent glucocorticoid treatments, and underlying comorbidities associated with T2D clearly can play a role in the immune response to infection, both locally, in the nasopharyngeal tract, and systemically in the lung and elsewhere (Bennett et al., 2021; Montefusco et al., 2021). However, it is complicated by other associated etiologic elements that often coexist in both T1D and T2D. For example, coagulopathies, endothelial dysfunction, underlying cardiovascular disease, and chronic inflammation with an impaired adaptive immune system all can be present in a single individual and raise the risk of subsequent morbidity and mortality (Chen et al., 2020). Nonetheless, hyperglycemia, chronic inflammatory markers, and T-cell dysfunction remain the three predominant signs in patients with T2D and hence are also likely to be major drivers of morbidity during SARS-CoV-2 infection.

### Pancreatic islet cell dysfunction

Beta cell damage can be caused by several distinct mechanisms in patients with acute COVID-19. These include: beta cell failure due to diminished beta cell mass and/or insufficient insulin secretion from failing beta cells, insulin resistance, and/or increased hepatic glucose production. The former has attracted significant interest in how beta cells function during SARS-CoV-2 infection. There remains an ongoing debate about whether ACE2, the predominant receptor for SARS-CoV-2, is present in islet cells (Drucker, 2021). There is more evidence that one of the accessory proteins for viral binding, TMPRSS2 is expressed on beta cells, and also in the blood vessels of the pancreas. Other putative coreceptors for SARS-CoV-2 have also been examined by RNAseq, both bulk and single cell, from human islet donors (Fignani et al., 2020). There is some evidence that the soluble form of the surface T-cell activation antigen CD26, which possesses dipeptidyl peptidase 4 (DPP-4) activity, can serve as a coreceptor for SARS-CoV-2. As an enzyme, DPP-4 degrades GLPs and GLP-1 receptor agonists, and DPP-4 inhibitors are used to treat T2D. Soluble DPP-4 is also an adipokine, but importantly CD26 is thought to be involved in the binding of SARS-CoV-2 through its S1 recombinant receptor-binding domain, aiding in viral entry (Lamers et al., 2011). Inhibition of DPP-4 activity to reduce the severity of COVID-19 has been under investigation, but there are little data to support its use at present, either prophylactically or during infection (Solerte et al., 2020).

### Insulin resistance

Hyperglycemia due to insulin resistance is characterized by hyperinsulinemia as the pancreatic beta cells are still functional, but attempt to overcome hyperglycemia by increasing insulin secretion. One of the drivers of insulin resistance is chronic inflammation, which is a persistent feature of the comorbidities associated with SARS-CoV-2 both from the infection and underlying metabolic dysfunction. In a cohort of 551 patients hospitalized for COVID-19 in Italy, the authors found that 46% of patients were hyperglycemic, whereas 27% were normoglycemic. Using clinical assays and continuous glucose monitoring in a subset of those patients, the authors detected altered glycometabolic control, with insulin resistance and an abnormal cytokine profile (Montefusco et al., 2021). Even normoglycemic individuals had evidence of insulin resistance and increased cytokine levels.

### Increased hepatic glucose production

Recently, Wan et al. reported that GP73, a Golgi protein induced by infections, was increased during recent SARS-CoV-2 infection (Wan et al., 2022). Furthermore, they showed that injection of recombinant GP73 raised blood glucose in mice within 15 min. To address the mechanistic basis of those findings, the authors treated primary mouse hepatocytes with rmGP73 and observed a dose- and time-dependent increase in glucose release in association with increased levels of intracellular cAMP, activation of protein kinase A (PKA) and expression of key gluconeogenic genes. These data provide clues that enhanced hepatic glucose production may be responsible for the dysglycemia of SARS-CoV-2 infection.

### Obesity as a contributing factor to inflammation and SARS-CoV-2 susceptibility

The underlying metabolic dysfunction associated with obesity includes in almost every case a decrease in the functionality of white adipose tissue, characterized by inflammation and reduced neutral lipid storage in adipocytes. This in turn results in the deposition of lipids in other tissues and triggers immune and vascular pathology. Ectopic deposition is frequently associated with the generation of secondary signaling lipids, such as ceramides, which are proinflammatory, proapoptotic, and confer further insulin resistance in their own right. Prevailing hypoxia in obese adipose tissue can lead to the activation of hypoxia-inducible factor transcription elements that can turn on profibrotic programs as well. Fibrosis is a widespread phenomenon in obese adipose tissue, leading to enhanced adipocyte necrosis with associated local and subclinical systemic inflammation. Further exasperating these inflammatory conditions are slower gastric motility and increased intestinal permeability, leading to enhanced endotoxin levels in circulation. Patients with obesity and T2D are frequently ‘primed’ with enhanced baseline inflammation. Several studies have highlighted the vastly synergistic proinflammatory properties of enhanced plasma endotoxin levels with viral components, particularly the SARS-CoV-2 spike protein. Finally, observations in the recent past have highlighted the ability of adipocytes and ‘adipocyte-like cells’ (such as the lipofibroblasts in the lung and stellate cells in the liver) to undergo a dedifferentiation event, thereby losing typical adipocyte markers, such as the master adipogenic transcription factor PPARγ, as well as one of its primary target genes, adiponectin (Sun et al., 2020). In the lung, lipofibroblasts morph into myofibroblasts during the infection, which in turn are likely culprits in the dramatically enhanced profibrotic environment seen during viral exposure. In this context, the potent antifibrotic and anti-inflammatory factor adiponectin is reduced, while the proinflammatory adipokine leptin is enhanced (Elrobaa and New, 2021). Many immune cells of the innate and adaptive immune system, including T cells, express leptin receptors, and may disproportionally respond to the viral challenge. Stabilizing cells in their ‘inactive’ adipocyte-like state through activation of the PPARγ pathway may prevent the emergence of myofibroblasts. PPARγ is antifibrotic, anti-inflammatory, and potently upregulates adiponectin. Leptin can also be reduced through the use of neutralizing antibodies, thereby curbing a disproportionate immune response. Both the PPARγ and the leptin pathway offer significant potential in the future, not only in the context of the acute viral response, but also in the context of the prolonged consequences associated with PASC (Elrobaa and New, 2021; Tanowitz et al., 2017).

### T-cell dysfunction in T2D, obesity, and SARS-CoV-2

T cells play a primary role in the acute and chronic response to viral infection by aiding antibody and cytokine production. Recent reports suggest that T-cell dysfunction and aberrant cytokine production occur in T2D as well as obesity and may be an underlying cause of both infection severity and length of time to full recovery from SARS-CoV-2. Nikolajczyk et al. compared cytokine profiles generated by negatively selecting bead-purified T cells (>95% pure by CD3^+^CD4^+^ flow cytometry) from adults (mean age 55) with normal body weight and HbA_1c_ (BMI ~22 kg/m^2^, HbA_1c_ <5.7%) and patients with prediabetes (HbA_1C_ 5.7–6.4%). The authors stimulated cells with CD3/CD28 beads for 40 hr and then quantified secreted cytokines in a 25-cytokine multiplex (Ip et al., 2016; Beste et al., 2014; Lau et al., 2012). IL-10 was produced in greater amounts from cells of lean/healthy donors, and there were differences in production of Th1/Th17 cytokines, that is, more CCL-20, TNFα, GM-CSF in prediabetes, and less IL-6/-12 were noted (Liu et al., 2022). These data are consistent with the tenet that metabolic status, independent of age, significantly shifts inflammatory profiles. It is not surprising that altered cytokines and T-cell profiles could tip the balance between an acute response that is beneficial for viral clearance or viral evasion versus a longer term chronic inflammatory response in patients with T2D. The early cytokine response may hold the key for limiting viral propagation for most COVID-19 patients who are asymptomatic or develop mild symptoms. It also may be important for defining the overall clinical course of the disease and of ‘long covid’.

In respect to the innate immune response, interferons are the first line of defense with acute SARS-CoV-2 infection, and are secreted following release of viral contents into the cytoplasm. Induction of interferon stimulatory genes, chemokines, and cytokines occurs after interferon secretion. The early response is orchestrated by airway epithelial and alveolar epithelial cells (ATII) as well as alveolar macrophages. A regulated and controlled release of cytokines and chemokines in the early phase of infection is not proinflammatory but is focused on viral clearance in the vast majority of patients (Song et al., 2020). However, in a small percent of patients, that response is dysfunctional and can lead to a dramatic release of cytokines and the clinical scenario of ‘cytokine storm’. There appears to be a dramatic TH1 cell response with increased secretion of IFN-γ, GM-CSF, and IL-6, and this is associated with a hyperinflammatory state, particularly CD4+ and CD8+ T-cell activation, with the latter more acutely driven in the lungs and other tissues. In a recent paper, among 30 patients with acute COVID-19 of varying severity, the number of circulating CD26+ cells decreased during the acute illness, particularly in those with severe disease (deKay et al., 2021). Ultimately, CD26+ cells rose in those patients that fully recovered but not in those with prolonged disease or death. CD26 cell number correlated with hospital-free days, and in those with mild disease, indicating that in contrast to a soluble form, membrane-bound CD26 might be associated with T-cell-specific immunity against SARS-CoV 2. It has been reported that CD26+ T cells secrete high levels of IL-17, which in turn amplifies cytokine production in patients with acute COVID-19, and in ‘cytokine storm’ syndrome. Hence, similar to other adipokines, DPP-4 may be both beneficial or harmful depending on the temporal nature of infection and response.

Lymphopenia is a common finding in moderately to severely ill COVID-19 patients, and both circulating CD4+ andCD8+ T cells suffer exhaustion and depletion while activated T cells persist in tissues. However, the story is much more complex than simple depletion and other studies have suggested that the T-cell response is heterogeneous (Ahmad et al., 2020a). Interestingly Su et al. reported that gastrointestinal symptoms compared with respiratory symptoms of PASC are associated with unique T-cell clonal and transcriptional dynamics (Su et al., 2022). Clearly, more studies of a longitudinal nature are needed, and these may also shed light on the T-cell profile in PASC and the relationship to metabolic dysfunction and T2D.

## Metabolic dysfunction and PASC

### Overview of epidemiology

**Figure 1.**
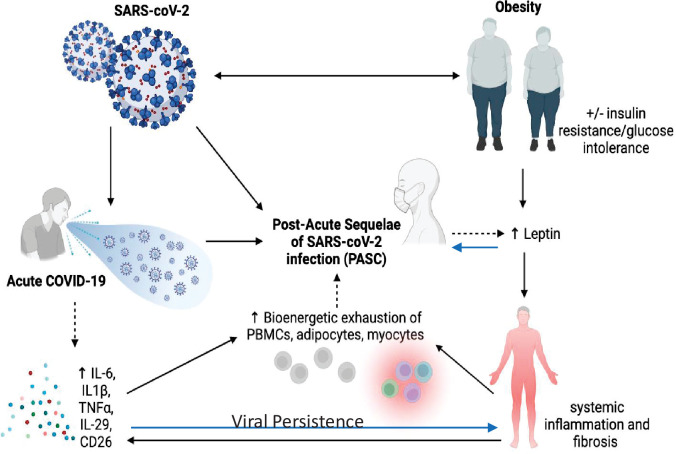
Pathophysiology of *Post-Acute Sequelae of CoV-2* (PASC) is multifactorial and may be exacerbated by insulin resistance and adiposity due to chronic inflammation and viral persistence.

It is almost certain that the underlying pathophysiology of PASC is multifactorial due to the plethora of sequelae, symptomology, and extent of tissue injury (A Sehgal et al., 2021; see Figure 1). Anecdotal data suggest PASC is more common in women than men, but no firm conclusions can be drawn yet. Similarly, it is unclear whether there are racial or ethnic differences in the prevalence of PASC, and whether vaccination status or viral variant influences post-COVID-19 symptomatology or prevalence. Nevertheless, there are multiple potential theories about the etiology of PASC. Viral persistence, either RNAemia or SARS-CoV-2 in tissues, ongoing immune responsiveness, reactivation of Ebstein–Barr virus, metabolic status, dysbiosis of the gut, autoimmune antibody development, and other factors all could contribute to the etiology of this syndrome. However, data are scarce and hence the evidence base is marginal and there are many confounders. For example, a recent review of studies up to mid-January 2022 showed that vaccinated individuals were far less likely to develop PASC, particularly those vaccinated within a week of their acute infection (UK Health Security Agency, 2022). However, due to other confounders, including length of follow-up after vaccination, the overall conclusions must be viewed with caution. In addition to the timing of symptoms, other factors such as hospitalization, duration of symptoms, pre-existing conditions, and medications must be considered in the context of risk prediction. Although there are more outstanding questions than evidence surrounding the risk and incidence of PASC, there is emerging evidence that three main symptoms stand out in the majority of cases: fatigue, cough, and anosmia/dysgeusia (Su et al., 2022). In each of these T2D has been very strongly associated with those symptoms (Su et al., 2022).

### Risk of developing PASC in obese and T2D patients

In this section, we will focus on evidence that obesity and metabolic dysfunction are linked to PASC. First, there are consistent data that background metabolic dysfunction in an individual predisposes to subsequent PASC. One study in Italian healthcare workers found obesity to be a major risk factor for PASC. In a recent *Cell* paper, the authors demonstrated that T2D and high levels of acute SARS-CoV-2 RNAemia were strong predisposing risk factors for PASC (Su et al., 2022; Vimercati et al., 2021). In another case–control study, patients with T2D who developed COVID-19 had a much greater likelihood of having high fatigue scores postinfection than those controls who were not infected (Mittal et al., 2021). Moreover, patients with T2D had poorer grip strength post-COVID, and increased markers of inflammation relative to noninfected controls (Su et al., 2022). On the other hand, in another study, diabetes was not a risk factor for experiencing long-term post-COVID symptoms. One hundred and forty five patients with diabetes and 144 control subjects without diabetes who had recovered from COVID-19, were assessed at 7.2 (SD 0.6) months after hospital discharge. The number of post-COVID symptoms was similar between groups (incident rate ratio 1.06, 95% confidence interval [CI] 0.92–1.24, p = 0.372). The most prevalent post-COVID symptoms were fatigue, dyspnea on exertion, and pain. No between-group differences in any post-COVID symptom were observed but the numbers were relatively small, and the upper CI clearly exceeded 1.0 (30).

### Risk of developing new onset T2D or insulin resistance

Several reports have supported the tenet that there is an increased frequency of T2D and new onset glucose intolerance post-COVID (Aminian et al., 2021; Leong et al., 2020; Su et al., 2022; Xie and Al-Aly, 2022; Maestre-Muñiz et al., 2021; see Table 1). In a retrospective electronic health record cohort study to identify phenotypes that positively associate with a past positive (RT-PCR(Reverse Transcription-polymerase chain reaction)) test for COVID-19, the authors found 33 phenotypes among different age/gender cohorts or time windows that were positively associated with past SARS-CoV-2 infection (Maestre-Muñiz et al., 2021). Two months or more after a COVID-19 RT-PCR test in nonhospitalized patients a new diagnosis was recorded for anosmia and dysgeusia (odds ratio [OR] 2.60, 95% CI [1.94–3.46]), chest pain (OR 1.27, 95% CI [1.09–1.48]), chronic fatigue syndrome (OR 2.60, 95% CI [1.22–2.10]), and T2D (OR 1.41, 95% CI [1.22–1.64]; Fernández-de-Las-Peñas et al., 2021). Moreover, a recent report from the CDC has raised additional concern about the development of diabetes after COVID-19 (Barrett et al., 2022). To estimate the risk for any new diabetes diagnosis (type 1, type 2, or other diabetes) >30 days after acute infection with SARS-CoV-2, the CDC estimated diabetes incidence among patients aged <18 years (patients) with diagnosed COVID-19 from retrospective cohorts constructed using IQVIA healthcare claims data from March 1, 2020 through February 26, 2021, and compared it with incidence among patients matched by age and sex who (1) did not receive a COVID-19 diagnosis during the pandemic, or (2) received a prepandemic non-COVID-19 acute respiratory infection diagnosis (Barrett et al., 2022). Analyses were replicated using a second data source (HealthVerity; March 1, 2020–June 28, 2021) that included patients who had any healthcare encounter possibly related to COVID-19. Among these patients, diabetes incidence was significantly higher among those with COVID-19 than among those: (1) without COVID-19 in both databases (HR IQVIA data: 2.66 [1.98–3.56], HR Health Verity: 1.31 [1.2–1.44]), and (2) with non-COVID-19 acute respiratory infections in the prepandemic period HR: 2.16 (1.64–2.86). However, these data were not adjusted for obesity, medications, race, or ethnicity (Barrett et al., 2022). In addition, these are claims reports which are notorious for confounding factors. Nonetheless, there are clues that COVID-19 could result in late onset diabetes, particularly in individuals that have borderline diabetes to start out with. In a longitudinal follow-up of 736 patients with a positive COVID-19 diagnosis early in the pandemic, in Spain, new onset diabetes was diagnosed at 1 year post-COVID-19 recovery in 7 patients (1.3%). In addition, intensification of oral antidiabetic drugs or insulin was required in 15 previously known diabetic patients (of which there were 189 patients). Two others with diabetes were diagnosed with peripheral neuropathy, and three more with retinopathy, after recovery from acute COVID-19 (Xie and Al-Aly, 2022). In another study, Montefusco et al. reported that among 551 patients without a history of diabetes and normal HbA_1c_, nearly half had glycemic abnormalities during the acute illness, and these changes could be detected for at least 2 months in patients who recovered from COVID-19 (Montefusco et al., 2021). Taken together, most of the evidence suggests that glucose intolerance persists or may even become clinically apparent post-COVID-19, and that both T1D and T2D patients are at risk of worsening disease.

### Underlying metabolic clues to the pathophysiology of PASC

1. Systemic viral persistence in PASC. There is emerging evidence that SARS-CoV-2 can infect and persist in adipose tissue and elsewhere (Martínez-Colón et al., 2021; Reiterer et al., 2021). Li et al. have published extensively on both viral persistence and viremia during COVID-19 (Fajnzylber et al., 2020; Choi et al., 2020; Li et al., 2021). This group has demonstrated that SARS-CoV-2 dissemination is commonly detected and that the magnitude of plasma viral load is associated with lower absolute lymphocyte counts, increased markers of inflammation (including C-reactive protein and IL-6), and a worse prognosis (Fajnzylber et al., 2020). The source for the plasma viremia is still not fully defined and could reflect spillage from the pulmonary tissue into the vasculature, but there is evidence that SARS-CoV-2 can also directly infect endothelial cells (Fajnzylber et al., 2020). Tissue studies have revealed evidence of endothelitis with perivascular inflammation and the extrapulmonary spread of SARS-CoV-2 to other organs (Caccuri et al., 2021; Elrobaa and New, 2021). Some clues from a case study suggest that in at least a subset of patients, the extrapulmonary burden of SARS-CoV-2 may be attributed to a substantial fraction of the virus found in the body (Li et al., 2021). In addition, a case report from an autopsy of a young individual acutely infected with SARS-CoV-2 detected the presence of spike protein in intestinal cells (Mayordomo-Colunga et al., 2021). Similarly, it is established that the virus can be detected in feces, and possibly spread through fecal contamination (Moura et al., 2022), although there are no data yet as to whether there is persistent viral shedding in long-COVID patients. What is certain is that gastrointestinal symptoms persist in PASC and that there is significant disruption of the intestinal microbiome from acute and possibly chronic infection with SARS-CoV-2 (Roy et al., 2021).

2. Viral persistence in adipose tissue. Importantly ,recent reports have demonstrated the presence of SARS-CoV-2 virus in human adipose tissue and the long-term presence of SARS-CoV-2 RNA in tissue samples that lead to evolution of virus-specific antibodies (Fajnzylber et al., 2020; Zickler et al., 2022). Martinez-Colon et al. identified two cellular targets of SARS-CoV-2 infection in adipose tissue mature adipocytes in adipose depots and macrophages. Mature adipocyte viral RNA was noted from several tissues, including visceral and epicardial samples, in an autopsy series (Reiterer et al., 2021). SARS-CoV-2 was found in at least one adipose tissue depot in 10 of the 18 male individuals (Zickler et al., 2022). Yet, the virus was found only in adipose tissue of the male individuals who were overweight (BMI >25) or obese (Zickler et al., 2022). On the other hand, adipose tissue macrophage infection is largely restricted to a highly inflammatory subpopulation of macrophages, present at baseline, that is further activated in response to SARS-CoV-2 infection. Preadipocytes, while not infected, adopt a proinflammatory phenotype. In addition SARS-CoV-2 RNA is detectable in adipocytes in COVID-19 autopsy cases and is associated with an inflammatory infiltrate. Remarkably, studies in hamsters and humans suggest that SARS-CoV-2 suppresses de novo lipogenesis as well as adiponectin and adipsin expression in mature adipocytes (Reiterer et al., 2021; Zickler et al., 2022). Both of these phenomena are typically associated with dysfunctional adipose tissue and T2D. It is untested whether patients with PASC clear viral particles from their adipose tissue more slowly or maintain an active immune clearance mechanism or for that matter have viral persistence. Given the very long half-life of human adipocytes (up to 10 years), adipocytes can serve as a reservoir for long-term presence of a number of parasites and viral particles (Tanowitz et al., 2017).

3. Chronic inflammation, PASC, insulin resistance, and viral persistence. Patients with obesity and T2D are frequently ‘primed’ by enhanced baseline inflammation which can be linked to clinical symptomatology (Su et al., 2022). There are certain driving forces that make the background of obesity a fertile ground for persistent inflammation in the face of RNAemia or auto-antibody signaling. Adipocyte dysfunction due to expansion of the lipid droplet can lead to ectopic lipid accumulation and release of lipid signaling factors that drive inflammation and also lead to enhanced insulin resistance, superimposed on beta cell failure or increased hepatic glucose production. Pre-existing fibrosis and apoptosis can perpetuate inflammatory responses further in individuals with obesity in response to viral persistence in adipose tissue. In addition, patients with T2D can have enhanced intestinal permeability leading to endotoxemia and progressive inflammatory responses from T cells, dendritic cells, and fibroblasts. Therefore, there is good reason to believe that all these factors combined are responsible for a more severe progression of the disease in the acute setting. Furthermore, the following may be key players in the setting of PASC: (1) the multitude of different immune cells present in adipose tissue that can switch into more aggressive proinflammatory subtypes over the course of infection; (2) the dysfunctional adipocyte that can act as a master regulator of the proinflammatory response in adipose tissue as a whole, which is primed for more severe inflammatory responses, including but not limited to the action of TLR-4 (the endotoxin receptor) on the cell surface of the adipocyte (Wernstedt Asterholm et al., 2014); (3) the fact that the adipocyte can be a target for infection by SARS-CoV-2 and that the infection may persist in adipocytes for prolonged periods of time (although this still has to be formally proven); (4) the ability of adipocytes to dedifferentiate into myofibroblasts that extensively contribute an overall profibrotic microenvironment; (5) the dysfunctional adipocyte can be center stage (and in many cases ground zero) for local and systemic insulin resistance and ensuing hyperglycemia. Still questions remain about the adipocyte and SARS-CoV-2 infection. For example, are there differences in the extent of viral persistence by adipose depot? Do racial or ethnic differences determine greater or lesser inflammatory responses to viral infections, short or long term? Does sex impact the prevalence of PASC and is this related to differences in adipose distribution?

### Summary and challenges ahead for treating PASC

Although there are accumulating data that adipose tissue dysfunction and hyperglycemia play a significant role in the clinical course of SARS-CoV-2 infection, there remain huge gaps in our knowledge base, particularly in relation to the etiology, epidemiology, and treatment of PASC. Importantly, one of the biggest unknowns is whether SARS-CoV-2 can persist in islet cells and whether insulin deficiency, either due to viral destruction, or local T-cell infiltration is present. Hence, it is difficult to propose therapeutic regimens that treat or shorten ‘long-COVID’ from a metabolic perspective. Longitudinal studies of glucose tolerance and insulin sensitivity will be required among PASC individuals to assess the distinction between insulin resistance and deficiency. Notwithstanding, based on numerous observations, adipose tissue may be a direct target for intervention through the use of a broad range of antidiabetic agents that lead to improvements in systemic metabolic profiles which might prove beneficial in other ways as well (Sun et al., 2020). Among the various choices, treatments that lead to an enhanced activity of PPARγ seem to be particularly promising (Sun et al., 2020) for the reasons outlined within, as these agents target every one of the phenomena listed from (A) through (E) Colca and Scherer, 2022. Generic anti-inflammatories, such as dexamethasone, are already in widespread use, but alternative, more targeted interventions may be appropriate as well. This includes in particular the proinflammatory cytokine leptin whose plasma levels can be effectively reduced with neutralizing antibodies. The biggest challenge we are facing is the lack of insight as to whether any of these interventions bear promise for the long-term consequences seen in PASC. However, we expect much progress in this area over the next 6–12 months when the full extent of the magnitude of the problem will become clearer, the pathophysiology better understood, and the clinical protocols for treatment more established.

**Table 1.** Risk of new onset metabolic dysfunction (T2D/hyperglycemia for post-SARS-CoV-2 patients).

Author	Study type	HR or OR (95% CI), comments
Aminian et al	Longitudinal COVID + 2800 pts	1.39 (1.13–1.71), for future Dx testing
Leong et al	Mendelian Random UKBio	1.14 (1.07–1.21), BMI and COVID-19 + severe
Xie et al	Cohort Study 180,000 VA Records	1.40 (1.36–1.44), incident DM of COVID-19+
Fernández-de-Las-Peñas	Case Control 299	1.06 (0.92–1.24), not significant DM risk
Barrett et al.	Two Retrospective Cohorts Record IQVIA, HealthVerity Covid+	2.66 (1.98–356) for DM (claims data)*1.31 (1.20–1.44) for DM (claims data)
Montefusco	Prospective 551 pts hospitalized	46% of COVID-19 + pts hyperglyc at 2 months

* CDC MMWR report.
